# *CYP2D6* Reduced Function Variants and Genotype/Phenotype Translations of CYP2D6 Intermediate Metabolizers: Implications for Personalized Drug Dosing in Psychiatry

**DOI:** 10.3389/fphar.2021.650750

**Published:** 2021-04-22

**Authors:** Espen Molden, Marin M. Jukić

**Affiliations:** ^1^ Center for Psychopharmacology, Diakonhjemmet Hospital, Oslo, Norway; ^2^ Section for Pharmacology and Pharmaceutical Biosciences, Departement of Pharmacy, University of Oslo, Oslo, Norway; ^3^ Section of Pharmacogenetics, Department of Physiology and Pharmacology, Biomedicum 5B, Karolinska Institutet, Stockholm, Sweden; ^4^ Department of Physiology, Faculty of Pharmacy, University of Belgrade, Belgrade, Serbia

**Keywords:** CYP2D6, CYP2D6*9, CYP2D6*10, CYP2D6*41, intermediate metabolizer, activity score, pharmacogenetics

## Abstract

Genetic differences in cytochrome P450 (CYP)-mediated metabolism have been known for several decades. The clinically most important polymorphic CYP enzyme is CYP2D6, which plays a key role in the metabolism of many antidepressants and antipsychotics, along with a range of non-psychiatric medications. Dose individualization based on *CYP2D6* genotype to improve the effect and safety of drug treatment has been an ambition for a long time. Clinical use of *CYP2D6* genotyping is steadily increasing; however, for pre-emptive genotyping to be successful in predicting individual dose requirements, high precision of genotype-to-phenotype translations are required. Recently, guidelines for assigning CYP2D6 enzyme activity scores of *CYP2D6* variant alleles, and subsequent diplotype-to-phenotype translations, were published by the Clinical Pharmacogenetics Implementation Consortium (CPIC) and the Dutch Pharmacogenetics Working Group. Consensus on assigning activity scores of *CYP2D6* variant alleles and translating diplotype scores into CYP2D6 poor, intermediate, normal, or ultrarapid metabolizer groups were obtained by consulting 37 international experts. While assigning enzyme activities of non-functional (score 0) and fully functional (score 1) alleles are straightforward, reduced function variant alleles are more complex. In this article, we present data showing that the assigned activity scores of reduced function variant alleles in current guidelines are not of sufficient precision; especially not for CYP2D6*41, where the guideline activity score is 0.5 compared to 0.05–0.15 in pharmacogenetic studies. Due to these discrepancies, *CYP2D6* genotypes with similar guidelinediplotype scores exhibit substantial differences in CYP2D6 metabolizer phenotypes. Thus, it is important that the guidelines are updated to be valid in predicting individual dose requirements of psychiatric drugs and others metabolized by CYP2D6.

## Introduction

The polymorphic enzyme CYP2D6 plays a key role in the metabolism of around 25% of all clinically used drugs, among which many are used in treatment of psychiatric diseases (Ingelman-Sundberg, 2005; Milosavljevic et al., 2020; van Schaik et al., 2020; van Westrhenen et al., 2020). CYP2D6 metabolizer phenotype is highly dependent on *CYP2D6* genotype (Bradford, 2002; Ingelman-Sundberg, 2005; Gaedigk et al., 2008; Caudle et al., 2020), which was recently shown to significantly predict therapeutic failure of the antipsychotic CYP2D6 drug substrate risperidone in a cohort of 1,288 patients (Jukić et al., 2019). This large-scale study shows that there is a potential of personalized dosing of CYP2D6 drug substrates based on *CYP2D6* genotype, and hence improve clinical outcome of many psychiatric drugs.

For the clinical utility, however, it is essential to provide accurate *CYP2D6* genotype-to-phenotype translations. Patients are traditionally allocated to four different CYP2D6-metabolizer phenotype subgroups: (1) poor metabolizer (PM), exhibiting absent enzyme activity; (2) intermediate metabolizer (IM), exhibiting reduced CYP2D6 enzyme activity; (3) normal metabolizer (NM), exhibiting fully functional CYP2D6 enzyme activity; and (4) ultra-rapid metabolizer (UM), exhibiting enzyme increased CYP2D6 enzyme activity. Since more than 140 *CYP2D6* variant alleles have been reported (www.pharmvar.org/gene/CYP2D6; accessed December 2020), encoding either no, reduced, fully functional or increased CYP2D6 metabolism, genotype-to-phenotype translations into the four metabolizer phenotype subgroups may be complicated.

More than 10-years ago a system for assigning enzyme activity scores of the various *CYP2D6* variant alleles was established by Gaedigk and colleagues to standardize genotype-to-phenotype translations (Gaedigk et al., 2018). In this system, *CYP2D6* variant alleles are assigned enzyme activity scores between 0 and 1, referring to no (‘*Null*’) and fully functional enzyme activities, respectively, and reduction-function variants with a score of 0.5. Based on the assigned activity score of the alleles, individual diplotypes scores are calculated ranging from ‘0’ for *Null/Null* carriers to ‘≥3’ for carriers of multiplications of fully functional alleles (Gaedigk et al., 2008; Gaedigk, 2013; Hicks et al., 2017; Caudle et al., 2020). The concept of the CYP2D6 ‘activity score’ has gained acceptance as a simple tool for CYP2D6 genotype/phenotype translations, and been adopted by the Clinical Pharmacogenetics Implementation Consortium (CPIC), and later by the Dutch Pharmacogenetics Working Group (DPWG), with the intention to facilitate the application of pharmacogenetics knowledge into clinical care.

While translating diplotype activity scores of *CYP2D6 Null/Null* (AS = 0) and CYP2D6*1/*1 (AS = 2) into CYP2D6 PM and NM phenotypes is straightforward, the diplotype score translations into the heterogeneous IM subgroup are complex, relying on the assigned activity scores of the reduced function variant alleles, which to some extent have differed between CPIC and DPWG. Thus, to standardize the *CYP2D6* genotype/phenotype translations, CPIC and DPWG recently published consensus guidelines on the assignment of activity scores of specific variant alleles for diplotype-based allocations into CYP2D6-metabolizer groups. In the new guidelines, the most relevant reduced function variants were assigned activity scores of 0.25 or 0.5.

The following diplotype score-to-phenotype translations are defined by the consensus guidelines: i) score 0 to PM, ii) scores >0 and ≤1.25 to IM, iii) scores >1.25 and ≤2.25 to NM, and iv) scores >2.25 to UM (Caudle et al., 2020). Furthermore, ‘PM spectrum’ was introduced as a new term referring to diplotype scores between 0 and ≤0.25. Accordingly, the assigned activity scores of reduced function variant alleles may determine the overall diplotype score, and hence the metabolizer-phenotype group allocation, phenotype ‘ranking’ within the heterogeneous IM group, and ultimately the precision of the *CYP2D6* genotype-to-phenotype translation.

In the literature, the activity scores of the specific reduced function variants are not consistently defined. Thus, *CYP2D6* genotype/phenotype translations of reduced function diplotypes allocated to the IM group by the guideline diplotypes may be uncertain. Furthermore, other factors than the *CYP2D6-*metabolizer phenotype determines drug clearance, which may limit the usefulness of the diplotype activity score in predicting individual exposure and dose requirements of CYP2D6 drug substrates. This article provides perspectives on these issues aiming to:

(1) critically assess the assigned activity scores of reduced function *CYP2D6* variants in current guidelines vs. human *in vivo* pharmacogenetic studies in relation to defining intermediate metabolizers.
(2) evaluate the CYP2D6 activity score model as a tool for predicting exposure and personalized dose requirements of drugs metabolized by CYP2D6.
(3) discuss future directions for *CYP2D6* genotype-based algorithms in predicting individual dose requirements of CYP2D6 drug substrates.

## Subsections

### Assigned Activity Scores of CYP2D6 Reduced Function Variant Alleles in Current Guidelines

In the guidelines from CPIC and DPWG, consensus on activity score assignments of specific *CYP2D6* variant alleles and genotype-to-phenotype translations were reached via interviews/opinions of 37 experts by the Delphi survey technique (Caudle et al., 2020). The most studied *CYP2D6* reduced function variant are CYP2D6*9, CYP2D6*10 and CYP2D6*41. The activity scores of CYP2D6*9 and CYP2D6*41 were defined as 0.5, while CYP2D6*10 was assigned an activity score of 0.25.

Interestingly, 44% of the members of the consensus group voted for defining diplotype scores ≤0.25 as *p*M. This was the basis for describing diplotypes scores between 0 and 0.25 to be in the ‘PM spectrum’ on the proposed linear activity score continuum. The latter is probably the major consequence of differentiating the activity score of CYP2D6*10 from CYP2D6*9 and CYP2D6*41. CYP2D6*10/Null carriers then are allocated in the ‘PM spectrum’ (score ≤25%), while CYP2D6*9/Null and CYP2D6*41/Null carriers are translated into the ‘pure’ IM group along with homozygous carriers of *CYP2D6* reduced function alleles and CYP2D6*1/Null carriers. Similarly, CYP2D6*1/*10 carriers (AS 1.25) are allocated to the IM group, while CYP2D6*1/*9 and CYP2D6*1/*41 carriers are defined within the NM group. Thus, the assigned activity scores of CYP2D6*10 vs. CYP2D6*9 and CYP2D6*41 is the most critical point of the genotype-to-phenotype translation in current guidelines, which should be compliant with reported activity scores in human pharmacogenetic studies on CYP2D6 substrates.

### Activity Scores of CYP2D6 Reduced Function Variant Alleles in Pharmacogenetic Studies

In the literature, there are several well-powered *in vivo* pharmacokinetic/genetic studies with a range of different CYP2D6 drug substrates where the CYP2D6 activity scores of reduced function variants can be calculated. The activity score (AS) of the respective reduced function variant (X) can be calculated using the following formula based on metabolic ratios (MR) of the CYP2D6 drug substrates in different genotype subgroups (Haslemo et al., 2019):

AS of X=(MR X/Null−MR Null/Null):(MR Wt/Null−MR Null/Null)

[Null alleles comprising CYP2D6*3, CYP2D6*4, CYP2D6*5 and CYP2D6*6, while absence of detected variant alleles defining Wild type (Wt; CYP2D6*1)].

Using this formula, we calculated the CYP2D6 residual enzyme activity scores of the most relevant reduction-function variants (CYP2D6*9, CYP2D6*10 and CYP2D6*41) against the metabolism of different CYP2D6 substrates (Table 1). In the Table, some data on the reduced function allele CYP2D6*17, which is frequent in Africans and Afro-Africans, are also presented.

**TABLE 1:** The most relevant reduced function variants with the respective mutations and frequencies in different ethnic groups, and activity scores defined by the CPIC/DPWG consensus guidelines vs. activity scores calculated from pharmacogenetic studies with various CYP2D6 substrates.

Variant	Variant frequencies, %			Activity scores		CYP2D6 substrate	Population	Participants, n	References
	Europe	Asia	African	CPIC/DPWG	Reported				
CYP2D6*9	2	<0.5	<0.5	0.5	0.34^a^	Venlafaxine	Norwegian	1,003	Haslemo et al. (2019)
					0.18^a^	Risperidone	Norwegian	1,318	Jukić et al. (2019)
					0.28^a^	Aripiprazole	Norwegian	1,437	Jukić et al. (2019)
					0.22	Vortioxetine	Mixed	1,140	Frederiksen et al. (2020)
CYP2D6*10	3	40	5	0.25	0.34^a^	Venlafaxine	Norwegian	1,003	Haslemo et al. (2019)
					0.18^a^	Risperidone	Norwegian	1,318	Jukić et al. (2019)
					0.28^a^	Aripiprazole	Norwegian	1,437	Jukić et al. (2019)
					0.37	Vortioxetine	Mixed	1,140	Frederiksen et al. (2020)
					0.22	Dextromethorphan	Japanese	162	Kubota et al. (2000)
					0.10	Dextromethorphan	Japanese	98	Tateishi et al. (1999)
CYP2D6*17	<0.5	<0.5	20	0.5	0.17	Vortioxetine	Mixed	1,140	Frederiksen et al. (2020)
					0.54	Metoprolol	Tanzanian	35	Wennerholm et al. (2002)
					0.09	Dextromethorphan	Tanzanian	35	Wennerholm et al. (2002)
					0.46	Codeine	Tanzanian	35	Wennerholm et al. (2002)
					0.12	Debrisoquine	Tanzanian	35	Wennerholm et al. (2002)
CYP2D6*41	8	<0.5	3	0.5	0.09	Venlafaxine	Norwegian	1,003	Haslemo et al. (2019)
					0.04	Risperidone	Norwegian	1,318	Jukić et al. (2019)
					0.17	Aripiprazole	Norwegian	1,437	Jukić et al. (2019)
					0.21	Vortioxetine	Mixed	1,140	Frederiksen et al. (2020)
					0.12	Dextromethorphan	German	36	Abduljalil et al. (2010)
					0.10	Tamoxifen	Swedish	118	Thorén et al. (2020)

^a^ CYP2D6*9 and CYP2D6*10 merged.

Together, the studies used to calculate the activity scores comprise several thousands of patients (Table 1). Regardless of the substrate, the encoded CYP2D6 metabolism is unambiguously lower for CYP2D6*41 than for CYP2D6*9 and CYP2D6*10. For the typical CYP2D6 substrates dextromethorphan, risperidone, tamoxifen and venlafaxine, the reported enzyme activity score of CYP2D6*41 is between 0.04 and 0.12 compared to 0.5 in the CPIC/DPWG consensus guidelines. The activity scores of CYP2D6*9 and CYP2D6*10 are quite similar between different substrates and both variant alleles seem to encode an enzyme activity score of in the range 0.2–0.35, possibly with a somewhat lower relative activity of CYP2D6*10 in East-Asian than in Europeans populations (Table 1).

CYP2D6*17 is a common reduced function variant in African and Afro-African populations but rarely found in other ethnic groups. In a study by Wennerholm et al. (2002), the CYP2D6 enzyme activity of CYP2D6*17 was investigated in Tanzanians (*n* = 35) healthy volunteers after administration of the CYP2D6 substrates codeine, debrisoquine, dextromethorphan and metoprolol for at least seven days. A unique aspect of this study was that the enzyme activities of multiple CYP2D6 substrates could be calculated in the same subgroups (Wennerholm et al., 2002). A striking finding was that the enzyme activity score CYP2D6*17 toward debrisoquine and dextromethorphan metabolism was 0.10, while the activity scores were around 0.50 for CYP2D6-mediated metabolism of codeine and metoprolol. This shows that the impact of this variant on CYP2D6 metabolism may be substantially affected by the substrate, which complicates the *CYP2D6* genotype-to-phenotype translations.

The inconsistencies between the *CYPD6* activity scores of the reduced function variants CYP2D6*41 and CYP2D6*9 defined in the current guidelines and those estimated from *in vivo* pharmacokinetic/genetic studies have a great impact on the calculated activity scores of the respective diplotypes, and hence the CYP2D6 genotype-to-phenotype translations. This is particularly relevant in the IM group, where many diplotypes comprise reduced function variants. Thus, for the assigned enzyme activity scores in the guidelines to be valid, the CYP2D6 phenotypes translated from different genotypes with the same diplotype scores should be similar.

### CYP2D6 Metabolism in IM Patients with Different Genotypes and Similar Guideline Diplotype Scores

To evaluate the precision of the guideline-assigned *CYP2D6* diplotype scores in predicting CYP2D6 metabolizer phenotypes, we compared CYP2D6 metabolism in patients with different *CYP2D6* genotypes but similarly defined diplotype activity scores consistent with an IM phenotype according to the CPIC/DPWG guidelines. The diplotype-to-phenotype consensus translations were evaluated using data from previous studies on three psychiatric CYP2D6 drug substrates, i.e., venlafaxine, aripiprazole and risperidone (Haslemo et al., 2019; Jukić et al., 2019), where we had access to raw data from individual patients.

**FIGURE 1:**
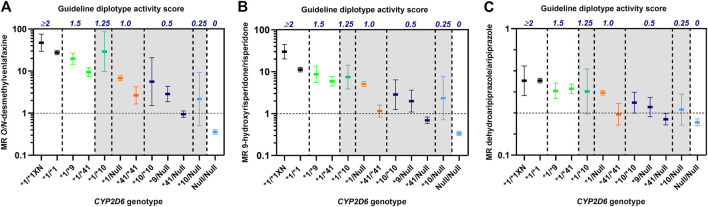
Metabolic ratios of *O/N*-desmethylvenlafaxine **(A)**, 9-hydroxyrisperidone/risperidone **(B)** and dehydroaripiprazole/aripiprazole **(C)** according to *CYP2D6* genotypes with similar diplotype activity scores as defined by the CPIC/DPWG consensus guidelines (Caudle et al., 2020). The vertical dashed lines indicate the spectra of different *CYP2D6* genotypes that are allocated to the same diplotype activity scores. The metabolic ratios, presented as geometric means with 95% confidence intervals, are based on data from publications by Haslemo et al., 2019 **(A)** and Jukić et al., 2019 **(B**, **C)**. Diplotype scores >0 to ≤1.25 (shaded) are by the consensus guidelines translated to CYP2D6 intermediate metabolizer (IM) group.

As shown in Figure 1 there were extensive differences in CYP2D6 metabolism toward venlafaxine, risperidone and aripiprazole between IM patients with similar *CYP2D6* diplotype scores, but different *CYP2D6* genotypes. One of the characteristic features was the consistently lower metabolism in CYP2D6*41/*41 carriers (diplotype score 1) than for the other IM-allocated genotypes with a diplotype guideline-score of 1 (Figure 1). Furthermore, the CYP2D6*41/*41 carriers (score 1) exhibited similar or lower metabolic ratios than CYP2D6*10/Null carriers (score 0.25) for all drugs (Figure 1). The CYP2D6*10/Null carriers are by the consensus guidelines proposed to exhibit CYP2D6-metabolizer phenotypes in the PM spectrum, but their metabolic ratios were substantially higher than for CYP2D6*41/Null carriers among patients treated with venlafaxine, risperidone and aripiprazole.

From the evidence provided from the above comparisons in big patient cohorts, it is clear that the assigned activity scores of the reduced function variants in the CPIC/DWPG guidelines do not comply with CYP2D6-metabolizer phenotypes observed among IM patients with similar *CYP2D6* diplotype activity scores originating from different *CYP2D6* genotypes. Thus, in clinical practice, the use of the current consensus guideline for diplotype-based *CYP2D6* genotype-to-phenotype translations does not predict individual CYP2D6-metabolizer phenotypes with sufficient precision. Instead of using the *CYP2D6* diplotype activity score as a surrogate measure of CYP2D6-mediated hepatic clearance, we propose applying the actual *CYP2D6* genotype to predict dose requirements of specific CYP2D6 drug substrates based on genotype-specific exposure in pharmacogenetic/kinetic studies. However, when predicting individual dose requirements of a specific CYP2D6 drug substrate, it is essential to account for other variables than the *CYP2D6* genotype that may determine its systemic exposure.

### Dose Predictions of CYP2D6 Drug Substrates Beyond CYP2D6 Genotype

As various CYP2D6 drug substrates exhibit different pharmacokinetic properties, it is necessary to account for other factors than *CYP2D6* genotype when predicting individual clearance values (Mehvar, 2018), and subsequently dose requirements of both psychiatric and other medications. While the *CYP2D6* genotype only predicts the partial intrinsic CYP2D6-mediated hepatic clearance (CL_int-2D6_), i.e. K_m_ and/or V_max_ values, the hepatic extraction ratio reflects the overall drug clearance in relation to hepatic blood flow (organ drug delivery) (Mehvar, 2018). For CYP2D6 drug substrates with a high extraction ratio (*E* > 0.7), blood flow rather than *CYP2D6* genotype (CL_int-2D6_) is the key variable determining individual hepatic clearance. However, for low-extraction drugs (*E* < 0.3), *CYP2D6* genotype (CL_int-2D6_) and free fraction in plasma determine hepatic clearance. Most drugs have a low or medium hepatic extraction ratio, implying that *CYP2D6* genotype (CL_int-2D6_) and free fraction in plasma are the main factors to take into account when predicting individual dose requirements of CYP2D6 drug substrates (Mehvar, 2018). In addition, body weight, kidney function etc. may be other relevant parameters physiological to take into account as integral parts of dosing algorithm.

Patients often use multiple medications concurrently, which imply that drug-drug interactions represent an issue that needs to be consider when predicting appropriate dosing of CYP2D6 drug substrates. For patients being genotype-predicted CYP2D6 NMs, phenoconversion to *p*Ms may occur during concomitant treatment with potent CYP2D6 inhibitors, e.g. the antidepressants bupropion, fluoxetine and paroxetine. A point is also that CYP2D6 substrates may be subjected to considerable metabolism via other enzymes, which is an aspect of importance for the sensitivity toward altered CYP2D6 activity. Thus, for genotype-based dose predictions of CYP2D6 drug substrates to be of relevance at all, it is necessary that CYP2D6 plays a major role in the overall metabolism/clearance of the drug. It is difficult to define a ‘major role’, but one may consider that CYP2D6 metabolism should be mediating at least 50% of the overall clearance for differences in CYP2D6 activity to be of general relevance. However, the exact relevance will differ between various CYP2D6 drug substrates.

## Discussion

We have in this article critically highlighted the assigned enzyme activity scores of the *CYP2D6* reduced function variants and the corresponding genotype-to-phenotype translations of the CYP2D6 IM group in the current CPIC/DPWG guidelines. A major issue is that the guideline-defined CYP2D6 enzyme activity score of the reduced function variant CYP2D6*41 is not compliant with the literature. It is crucial to update the consensus guidelines on this point, and it should be clarified that patients carrying the CYP2D6*41/Null genotype exhibit a CYP2D6-metabolizer phenotype close to the PM subgroup, which is evident from studies on many CYP2D6 drug substrates (Table 1; Figure 1).

The large inconsistencies in CYP2D6 metabolism between various IM genotypes with similar diplotype scores show the inadequacy of the consensus activity score model in predicting individual hepatic clearance and dose requirements of CYP2D6 drug substrates. These inconsistencies may have important clinical implications for genotype-based dose recommendations of many psychiatric drugs, as CYP2D6 generally plays a key role in the metabolism of such agents. However, the same concerns CYP2D6 drug substrates from other therapeutic classes, e.g., tamoxifen, where Thorén et al. (2020) reported a significantly stronger impact of CYP2D6*41 than CYP2D6*10 on the CYP2D6-mediated bioactivation to endoxifen (Thorén et al., 2020), which mainly mediates the preventive effect on breast cancer recurrence.

While the consensus guidelines define CYP2D6*41 with an activity score of 0.5, the published activity scores in pharmacogenetic studies on multiple CYP2D6 substrates are typically in the range 0.05–0.15 (Table 1). The CYP2D6*41 variant allele includes the *2988 G > A* polymorphism, which encodes a splicing defect reducing the CYP2D6 enzyme expression by around 90% according to *in vitro* studies (Raimundo et al., 2004; Toscano et al., 2006). Thus, by reducing the hepatic CYP2D6 levels (V_max_) the relative effects of on the CYP2D6-mediated Cl_int_ is expected to be approximately the same regardless of substrate.

While previously defining all reduced function variant alleles with an activity score of 0.5, the revised CPIC/DPWG consensus guidelines recently down-adjusted the activity score assignment of CYP2D6*10 to 0.25. Actually, an activity score of 0.25 for CYP2D6*10 is in line with the literature (Table 1). For CYP2D6*9, which has a low frequency across different ethnic groups, available *in vivo* evidence (Table 1; Figure 1) suggests an enzyme activity score of 0.25 as well. Patients carrying CYP2D6*9/Null or CYP2D6*10/Null could therefore be merged into a common diplotype-translated CYP2D6 IM phenotype (Figure 1). The data presented here on the CYP2D6 metabolism shown in CYP2D6*41/*41 carriers suggest that these patients can also be merged with CYP2D6*9/Null or CYP2D6*10/Null carriers in an IM subgroup in the lower end of the phenotype scale (Figure 1). According to the data shown in the present article, the diplotype score of CYP2D6*41/Null will be around 0.1, i.e. within the spectrum of the CYP2D6 PM phenotype (Figure 1).

Today, new marketed CYP2D6 drug substrates have results from pharmacogenetic studies that could be used for the development of *CYP2D6* genotype-based dosing algorithms in clinical practice. It is therefore important that medicines agencies and clinicians demand such data to be made available for the best possible prescription, and not to allow the option that such data may be kept unavailable by the manufacturer. There is an apparent conflict of interest in this regards, since for the manufacturer, for the marketing purposes, it is most favourable to have a ‘one-size-fits-all’ drug, for which is not necessary to adjust the dose individually, based on the procedures including genotyping.

The data presented here were from populations mainly comprising Caucasians. The important point is that there might be inter-ethnic differences in the impact of the same variant on the metabolism of CYP2D6 drug substrates. An example is CYP2D6*10, which may seem to have a greater effect on CYP2D6 metabolism in East Asians than in Europeans (Table 1). Thus, another aspect with regulatory demands, it that pharmacogenetic studies should be performed across ethnic groups. Finally, for CYP2D6 drug substrates with active metabolites, factors determining the clearance of the active metabolites also need to be accounted for when predicting dose requirements. An example reflecting the relevance of this aspect is risperidone, where renal function determines the subsequent clearance of the active metabolite 9-hyrdoxyrisperidone (paliperidone). Consequently, a risperidone-treated CYP2D6 IM patient with renal failure, where both risperidone and paliperidone are accumulated, obtains a substantially larger effect on the exposure of active moiety than predicted from the *CYP2D6* genotype itself.

In summary, we propose that genotype-to-phenotype translations of the heterogeneous IM group should be revised in the CPIC/DPWG guidelines; mainly due to the discrepancies between the guideline-assigned activity scores of *CYP2D6* reduced function alleles and those calculated from pharmacogenetic studies with multiple CYP2D6 drug substrates. In particular, it is critical to correct the guidelines on the assigned activity score of CYP2D6*41. Carriers of CYP2D6*41/Null diplotype obviously exhibit significantly lower CYP2D6-metabolizer phenotypes than carriers of the CYP2D6*9/Null or CYP2D6*10/Null diplotypes, and should rather be defined within the PM spectrum.

Furthermore, we have highlighted that different *CYP2D6* genotypes assigned the similar *CYP2D6* diplotype activity scores by the CPIC/DPWG consensus guidelines exhibit substantial differences in CYP2D6 metabolism. In this perspective, we propose leaving the simple *CYP2D6* diplotype activity score model and instead use the actual *CYP2D6* genotype as is - without any phenotype translations - in algorithms predicting individual dose requirements. Provided known effects of *CYP2D6* genotypes on the exposure of specific drugs, the measured *CYP2D6* genotype, as a proxy of the individual patient’s CL_int-2D6_, could be incorporated into drug-specific dose algorithms, where other significant, concentration-determining variables are integrated. Since many psychiatric drugs are metabolized by CYP2D6, development of such genotype-based dose algorithms may meet the expectations of personalized treatment of patients with psychiatric disorders.

## Data Availability

The data analyzed in this study is subject to the following licenses/restrictions: The de-identified datasets used for comparing CYP2D6 metabolism in patients with similar CYP2D6 diplotype scores have to be stored at the Center for Psychopharmacology, Diakonhjemmet Hospital, in line with the ethical approval for using the retrospective data from laboratory analyses without patient consent. However, anonymized data files can be shared on request if contacting the corresponding author. Requests to access these datasets should be directed to espen.molden@farmasi.uio.no.
